# Therapeutic Choices for Genitourinary Syndrome of Menopause (GSM) in Breast Cancer Survivors: A Systematic Review and Update

**DOI:** 10.3390/ph16040550

**Published:** 2023-04-06

**Authors:** Lucia Merlino, Giulia D’Ovidio, Viviana Matys, Maria Grazia Piccioni, Maria Grazia Porpora, Roberto Senatori, Maria Federica Viscardi, Antonio Vitale, Carlo Della Rocca, on behalf of Policlinico Umberto I Collaborators

**Affiliations:** 1 Department of Maternal, Infantile and Urological Sciences, University of Rome La Sapienza, Viale del Policlinico 155, 00161 Rome, Italy; 2 Italian Society of Colposcopy and Cervicovaginal Pathology (SICPCV), 00186 Rome, Italy; 3 Comprehensive Cancer Center, Fondazione Policlinico Universitario Agostino Gemelli IRCCS, Cattolica del Sacro Cuore University, Largo A. Gemelli 8, 00168 Rome, Italy; 4 Department of Medico-Surgical Sciences and Biotechnologies, Sapienza University, 04100 Latina, Italy

**Keywords:** genitourinary syndrome of menopause (GSM), breast cancer survivors (BCS), vulvovaginal atrophy (VVA), aromatase inhibitors (AI), vaginal lubricants, vaginal moisturizers, local hormone therapy, vaginal laser therapy

## Abstract

(1) Background: Genitourinary syndrome of menopause (GSM) is a medical condition that can affect breast cancer survivors (BCS). This is a complication that often can occur as a result of breast cancer treatment, causing symptoms such as vaginal dryness, itching, burning, dyspareunia, dysuria, pain, discomfort, and impairment of sexual function. BCS who experience these symptoms negatively impact multiple aspects of their quality of life to the point that some of them fail to complete adjuvant hormonal treatment; (2) Methods: In this systematic review of the literature, we have analyzed possible pharmacological and non-pharmacological treatments for GSM in BCS. We reviewed systemic hormone therapy, local hormone treatment with estrogens and androgens, the use of vaginal moisturizers and lubricants, ospemifene, and physical therapies such as radiofrequency, electroporation, and vaginal laser; (3) Results: The data available to date demonstrate that the aforementioned treatments are effective for the therapy of GSM and, in particular, vulvovaginal atrophy in BCS. Where possible, combination therapy often appears more useful than using a single line of treatment; (4) Conclusions: We analyzed the efficacy and safety data of each of these options for the treatment of GSM in BCS, emphasizing how often larger clinical trials with longer follow-ups are needed.

## 1. Introduction

Breast cancer represents a global issue in public health, ranking as the first most diagnosed cancer in women and the second most common cause of cancer-related death after lung and bronchial cancer [1].

According to data from GLOBOCAN 2020 provided by the International Agency for Research on Cancer (IARC), the incidence is growing worldwide with an estimated number of 2.261.419 new cases per year, while average mortality is constantly decreasing, accounting for 684.996 deaths, mainly thanks to the recent improvements in early detection techniques and the availability of novel therapeutic agents. [2]. According to the National Cancer Institute’s (NCI) definition, every patient who receives a diagnosis of breast cancer is a breast cancer survivor until the end of their lives [3].

For long-term survivors, special concerns about the quality of life and long-lasting side effects, including gynecologic issues, must be considered an increasingly important part of overall care [4].

Breast cancer usually occurs in people without any genetic predisposition (70–80%); however, 5–10% of cases are associated with hereditary syndromes [5,6].

It has been largely demonstrated that pathogenic mutations in the BRCA1 and BRCA2 genes increase the risk of developing breast cancer, respectively, by 65% and 45%, and are responsible for 90% of all hereditary BC cases [4].

Breast cancer is historically classified into four main subtypes: luminal A, luminal B, HER2 overexpression, and basal-like (also known as Triple Negative, TNBC) [7].

The above-mentioned biomarkers have strongly validated prognostic and predictive values, thus playing an essential role in the definition of the best treatment option [8].

However, the treatments used in the management of breast cancer are responsible for the development of GSM. In fact, it has been observed that about 70% of BCS present symptoms attributable to a GSM picture due to the hypoestrogenism they experience [9].

With the term genitourinary syndrome (GSM), we are going to indicate the set of signs and symptoms attributable to the genitourinary tract and caused by a lack of estrogens.

This leads to changes in the lower genitourinary tract, which is responsible for the signs and symptoms of GSM.

As for the symptoms relating to the genital component, we find dryness with poor lubrication, burning and irritation, and vaginal discharge; regarding the urinary component, we have dysuria, urinary urgency, and recurrent urinary infections. To this, we must add dyspareunia. These symptoms determine an important sexual dysfunction.

On physical examination, we can find labial atrophy, vaginal dryness, and clitoral atrophy. The appearance of the vaginal walls, observable with the speculum, may appear friable and hypopigmented; there may be traces of bleeding, which can already be observed upon insertion of the speculum [10].

Vulvovaginal atrophy (VVA) is an important component of GSM [11].

Several studies have shown that the aromatase inhibitor has a central role in determining the symptoms of menopause. In detail, there is evidence that it determines a severe VVA with dyspareunia, significant hot flashes, and musculoskeletal pain.

It is important to be aware of this because it is necessary to make an early diagnosis. The symptoms related to GSM are the cause of a significant decrease in quality of life.

Based on the type of hormonal treatment to which the woman is subjected, there is an increased risk of manifesting a form of secondary amenorrhea, characterized by the absence of previously regular menstruation for three months or previously irregular menstruation for at least six months. Lambertini et al. illustrate in-depth the assessment of the risk of infertility following the administration of the main anti-cancer therapies [12].

The guidelines for BC risk reduction suggest hormonal therapies that lead inevitably to early sterility and premature ovarian failure, which precede the early onset of secondary menopausal symptoms [13].

Chemotherapy has a gonadotoxic effect, causing the destruction of the ovarian follicles. The endocrine therapy used in the treatment of breast cancer causes inhibitory effects on both ovarian and endometrial functions [12].

Pre-menopausal women under therapy will inevitably undergo suppression of ovarian and endometrial function with subsequent manifestations of transient or permanent chemotherapy-induced amenorrhea. In particular, patients under treatment with aromatase inhibitors will be at an increased risk of developing GSM.

On the other hand, young women with a positive pathogenic mutation for BRCA 1 and 2 who agree to undergo preventive bilateral salpingo-oophorectomy will inevitably develop an early surgically induced menopause and a form of secondary permanent amenorrhea.

We are talking about a severe clinical condition that causes a reduction in the quality of life of such patients, so it is important to diagnose and treat it.

Unfortunately, this issue is often underestimated and undertreated, due to a lack of knowledge by health care specialists or due to a fear of BC recurrence and poor familiarity with the various available options. In fact, a study conducted in 2021 by Pearson et al., aiming to improve the understanding of health professionals’ knowledge and management of genitourinary symptoms in women with early breast cancer, by administering a survey to 144 oncology health professionals, demonstrated that most respondents recognized it as a common problem, but only 16% felt confident managing these symptoms and prescribing adequate therapies [14].

A similar study on 120 breast oncologists was conducted in 2017 by Biglia’s group in Turin: they assessed that, despite the fact that none of the physicians considered VVA a transient event or a secondary problem in BCS, only half of the oncologists (48%) directly illustrated VVA to the patients as a possible consequence. 41% of the oncologists referred BCSs to gynecologists to define VVA treatment, while 35.1% managed it alone [15].

Therefore, we aim to underline the importance of treating this condition in order to enhance BCS’s overall care and quality of life.

According to today’s knowledge, we have several therapy options:

- Hormonal, systemic, and topical treatments;
- Non-hormonal topical treatments;
- Physical therapy.

The gold standard for this pathology would be the use of estrogen, which is however contraindicated in women with BCS; in fact, the treatment is still the subject of discussion in this population.

According to current guidelines [16], first-line treatment is a non-hormonal therapy, such as moisturizing and lubricating vaginal creams, which appear to be useful in treating vaginal dryness and lead to an improvement in symptoms. If there is no response, local hormonal treatments are carried out: low-dose intravaginal ovules or intravaginal cream based on testosterone or DHEA. However, the problem is represented by estrogen-based creams and vaginal rings in subjects receiving aromatase inhibitor therapy because systemic absorption has also been demonstrated [16].

The revolution in this area is represented by physical therapies such as electroporation and radiofrequency with the possibility of conveying different active ingredients inside the tissues or even by laser therapy, which allows for improved tropism of the vulvovaginal mucosa.

The objective of this systematic review of the literature is to analyze the various types of treatment currently present with regard to GSM in BCS and understand how to combine them in the best way.

## 2. Materials and Methods

A systematic review of the literature on SGM management and therapeutic options in BCS was conducted on PubMed, Medline, and the Cochrane Library using the following search terms: genitourinary syndrome of menopause (GSM); breast cancer survivors (BCS); menopause; vulvovaginal atrophy (VVA); aromatase inhibitors (AI); vaginal lubricants; vaginal moisturizers; local hormone therapy; vaginal laser therapy.

Recommendations from international scholarly societies were also taken into account. The North American Menopause Society (NAMS) https://www.menopause.org (accessed on 24 February 2023); https://www.asco.org (accessed on 24 February 2023), the American Society of Clinical Oncology (ASCO); the International Menopause Society (IMS) https://www.imsociety.org (accessed on 24 February 2023); the Canadian Menopause Society https://www.sigmamenopause.com (accessed on 24 February 2023); the European Menopause and Andropause Society (EMAS) https://www.emas-online.org (accessed on 24 February 2023); the International Society for the Study of Women’s Sexual Health (ISSWSH) https://www.isswsh.org (accessed on 24 February 2023).

**Figure 1:**
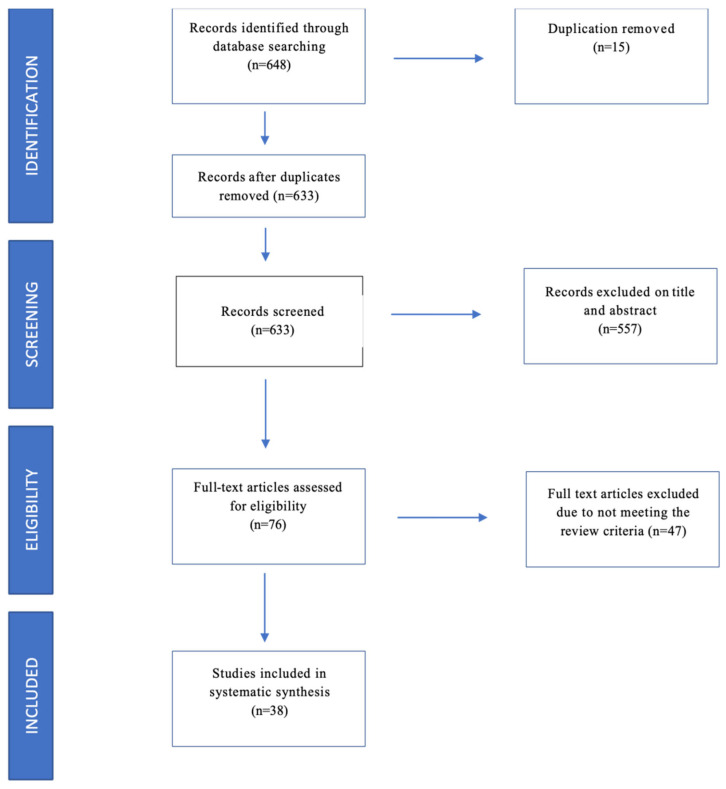
PRISMA flowchart [17].

Using PRISMA guidelines [17] for systematic review, we initially identified through database searches 648 records; after duplicates were removed, the records were 633; 577 of them were excluded based on the title and the abstract, so we assessed 76 full-text articles for eligibility. Full text excluded because it did not meet the review criteria was 47. Finally, we included in our systematic review 38 studies, all in English (Figure 1).

## 3. Discussion

The first-line treatment for vulvovaginal atrophy as well as for BCS is represented by non-hormonal therapies [18].

The group of non-hormonal therapies includes numerous categories of drugs and others that can play an optimal role in the treatment of pain during the sexual act and in general for vaginal well-being. Regarding GSM non-hormonal treatments in BCS, we have:

- Moisturizers and lubricants;
- Hyaluronic acid;
- Polynucleotides;
- Phytoestrogens;
- Vasodilators;
- Mechanical (dilators and sexual activity);
- Vaginal vitamin D and E;
- Vaginal/oral probiotics;
- Laser radiofrequency.

Moisturizers and lubricants: the role of vaginal moisturizers is to maintain the integrity and elasticity of the vagina; these are products that must be used regularly, therefore independently of sexual activity. Lubricants, on the other hand, are useful in reducing the sensation of discomfort during sexual acts. As far as lubricants are concerned, the WHO recommends using lubricants that have the following characteristics: osmolarity lower than 1200 mOsm/kg; this is because higher values are toxic and irritating to the vaginal mucosa. Glycerol content in the lubricant <9.9% mass fraction; propylene <8.3% mass fraction; if a glycol mixture is used, the limit must be less than 8.3% of the mass balance. The product must also be free from parabens, chlorhexidine, and polyquaternary compounds [19].

Hyaluronic acid (HA): linear polymer with large dimensions; it is flexible and extremely polar. It is a compound that we find to be the main component of the extracellular matrix (ECM), together with collagen, elastin, and fibronectin. It is one of the main constituents of the connective tissue of humans and mammals [20]. In the last 20 years, several studies have been carried out aimed at understanding the role of HA in both physiological and pathological conditions, and the biological mechanisms that regulate its synthesis, degradation, and metabolic activities have been analyzed. HA is synthesized on the inner surface of the plasma membrane and then extruded extracellularly; the synthesis is mediated by hyaluronic acid synthetase (HAS); we have 3 types of this enzyme with different functions: HAS1 and 2 polymerize HA of similar length, and HAS3 synthesizes HA with a short chain [21]. Long-chain HA has been shown to promote cell quiescence and only superficially support cellular integrity; low-molecular-weight HA instead promotes tissue repair. Taking into account what has been said, low molecular weight HA finds application both in the gynecological and urological fields. Several studies have shown that the topical use of ovules containing low molecular weight hyaluronic acid (LMW-HA) is a valid alternative in the short- and long-term treatment of problems such as itching, burning, dyspareunia, and dryness, symptoms therefore caused by alterations in the vaginal mucosa resulting both from treatments such as laser therapy, cryotherapy, and radiotherapy and from a lack of estrogen and therefore a GSM [22].

Polynucleotides: they are a mixture of low-molecular-weight fractions that form a linear polymer of deoxyribonucleotides joined by phosphodiester bonds. These are molecules capable of activating fibroblasts by activating purinergic receptors; they also stimulate growth by acting on CD39 receptors [23].

Phytoestrogens: they are substances of vegetable nature that are non-steroids, able to bind to estrogen receptors but with a weaker effect, from 100 to 1000 times a day. They also have antioxidant, anti-inflammatory, and antihypertensive properties. In the phytoestrogen family, isoflavones are of particular interest in the treatment of menopause, in particular genistein, contained in soy. Although the information and studies available are still very few, it has been seen that this molecule could be useful in the management of menopause problems both in the short and long term and therefore could also find utility in the treatment of GSM in BCS women [24].

Vasodilators: the extractive pyranocoumarin is a molecule with a vasodilating action. It interacts with the type 1 calcium channels at the level of the smooth muscles, causing an increase in the local flow. It has been observed that the local administration of a spray based on visnadine 10 minutes before sexual intercourse has a positive effect, so it is also a candidate among the non-hormonal alternatives to be used in the treatment of GSM in BCS [25].

Vaginal vitamin D and E: a study was conducted to understand the effect of vaginal suppositories based on vitamin E and D on vaginal atrophy in BCS. It is a randomized controlled trial. The result was an improvement in vaginal atrophy by administering these vaginal suppositories every night for 8 weeks [26].

Vaginal/oral probiotics: the vaginal microbiota (in detail, Lactobacillus spp.) plays a very important role in the health of the lower genitourinary tract, and a decrease has been observed during menopause. It has been observed that postmenopausal women with a vaginal microbiota dominated by Gardnerella vaginalis and low Lactobacillus develop vaginal atrophy much more easily than postmenopausal women with a microbiota high in Lactobacillus [27]. The administration of probiotics orally or vaginally is still the subject of a strong debate today, but on the basis of what they report, it certainly finds its usefulness.

Platelet-rich plasma: although there are few studies in the literature to date, it has been deduced that the use of platelet-rich plasma in the treatment of GSM, both as a single treatment and as an adjuvant, appears to be promising and has a good safety profile [28].

Mechanical (dilators and sexual activity): the use of dilators has been shown to support and help women suffering from genital pelvic pain and penetration disorders, which can be present in women with GSM [29].

The main problem with these substances, however, is that they are not able to regenerate the vaginal barriers or improve their characteristics. They can only slow down the evolution of the pathological picture, and the improvements obtained tend to be lost quickly after suspension. These are generally creaming moisturizers or lubricating gels composed mainly of water, vegetable oils, or silicone derivatives [30].

The main advantages related to non-hormonal therapy instead are represented by having practically no side effects; consequently, they can be used for long periods of time without the need for suspension periods. On the other hand, long periods are necessary for these treatments to start to take effect.

All non-hormonal therapies used in GSM therapy for breast cancer patients are summarized in Table 1.

From an etiopathogenic point of view, we know that the cause of vaginal atrophy, even in BCS patients, is caused by a drop in estrogen levels; consequently, a therapeutic treatment based on hormonal supplementation would seem logical. Local estrogen administration has been shown to be the most effective method. The effect consists of an improvement in the tropism of the vaginal mucous membranes and in tissue regeneration with the formation of new vessels and an increase in cell layers with the restoration of adequate local flora and an adequate pH [31].

The methods of administration of estrogens are numerous; there are solutions in cream, vaginal rings, ovules, or gels. The estrogen that is most frequently used in the treatment of vaginal atrophy is estriol.

Conversely, other recent prospective studies have suggested that the use of vaginal estrogen therapy may increase serum estrogen levels, resulting in a possible increased risk of BC recurrence. Santen et al. reported that an increase in serum estradiol levels produced by vaginal estrogens may not exceed the normal range of postmenopausal serum estradiol [32]. Some works in the literature suggest the use of estriol instead of estradiol for BCS since its metabolic clearance is faster. Estriol is not FDA-approved for any indication and should be used as an off-label hormone option [33]. Surely other studies are needed to validate the safety of hormone therapy with clear certainty, considering that the vaginal walls are extremely vascularized and that the use of local therapy does not exclude with certainty the entry into the circulation of hormones. Surely, from our point of view, we would like to specify that a case-by-case evaluation is necessary.

The administration of HRT should be limited to topical use; in fact, international guidelines do not recommend systemic administration [34].

Promestriene (3-propyl ethyl, 17β-methyl estradiol) is a synthetic estrogen analog with minimal systemic absorption for the topical treatment of vaginal atrophy, which can be used in BCF patients due to its poor activity on the breast. Studies by mass spectrometry have confirmed the low systemic activity even after months of therapeutic doses in women with high-grade estrogen receptor-positive breast tumors [35]. However, in vitro studies concluded that the potential estrogen-like properties of promestriene to stimulate the growth of estrogen receptor-reactive breast cancer cell lines, especially under conditions of estrogen deprivation, suggest caution when prescribing for vaginal atrophy in postmenopausal BCS. Its ability to activate growth and gene expression in ER-BC cells deserves further study.

Women who underwent surgery for luminal early breast cancer are all candidates to receive endocrine adjuvant systemic treatment, which was clearly related to a significant improvement both in progression-free survival (PFS) and overall survival (OS) [36,37].

In ER and PgR-positive BC, estrogens induce the proliferation of tumor cells and support the progression of the disease. Hormone therapy is administered in order to decrease the levels of circulating estrogens by using drugs active on the hypothalamic-pituitary-ovarian axis and the estrogen receptors [38,39].

Mechanisms and sites of synthesis of sexual hormones change over a woman’s life depending on reproductive age. In pre-menopausal women, estrogens are mainly produced by the granulosa cells of the ovary, whereas in post-menopausal women, the primary mechanism of production is the peripheral aromatization of C19-steroids by aromatase located in the adipose tissue [40].

Based upon these biological assumptions, pre- and post-menopausal women are candidates to receive different treatment options.

Two randomized trials, TEXT and SOFT, demonstrated that the use of LHRH-analogues (e.g., triptorelin, goserelin, and leuprorelin) in association with tamoxifen or exemestane resulted in a significant survival benefit for premenopausal women [41].

The ATAC and BIG 1–98 trials showed that in post-menopausal patients, monotherapy with aromatase inhibitors (e.g., anastrozole, letrozole) yielded better outcomes when compared with tamoxifen. In this group, the use of LHRH analogs is not recommended because ovarian production of estrogens is supposed to be physiologically abolished [42,43].

About 95% of patients exposed to endocrine therapy experience at least one drug-induced adverse event (AE), among whom 25–30% reach grade 3 or 4. The early discontinuation rate because of AEs is approximately 20%. Most common AEs include increased cardiovascular and thromboembolic risk, osteoporosis, and those issues that come under the name of genitourinary syndrome of menopause (GSM) [41].

Systemic loss of estrogen results in physiological and structural modifications within the genital structures and vaginal mucosa. Changes include reduced cervical gland secretions, deterioration of tissue, a decrease in blood flow, loss of elasticity, thinning of tissue and epithelium, and an increased pH. These changes are responsible for vaginal dryness and irritation, dyspareunia, decreased libido, frequent urinary tract infections, and urinary incontinence, which together are known as vulvovaginal atrophy or GSM [44,45].

All these symptoms have a great impact on quality of life and need to be properly recognized and managed by the treating physicians, often requiring a multidisciplinary approach. For a long time, this condition has gone undiagnosed and untreated due to a lack of awareness and the paucity of evidence about a safe and effective therapy. From an analysis of the literature and from daily clinical practice, it emerged that there is a lot of difficulty on the part of doctors in administering hormone replacement therapy in patients with previous breast cancer, due to the possible repercussions and side effects. The main fears are related to the risk of interfering with adjuvant therapy, thus favoring any relapses. Numerous works in the literature explain how the risk of tumor reactivation or recurrence is very low [46].

Currently, the cornerstone of treatment for GSM in patients receiving antiestrogen therapy is non-hormonal products. These kinds of solutions are not able to reverse atrophy once it has occurred but can help alleviate symptoms by increasing vaginal moisture, thus avoiding the use of any hormone-based compounds [47].

When non-hormonal products fail to control symptoms, estrogen-based therapy is the only effective option available.

The detrimental effect of systemic estrogen administration was established by two randomized trials. The HABITS trial enrolled 434 patients taking AIs with GSM and randomly assigned them to receive hormone therapy (estradiol hemihydrate and norethisterone) for 2 years or the best symptomatic treatment [48]. The Stockholm trial randomized 378 patients with the same characteristics to receive estradiol and medroxyprogesterone acetate or non-HT. Both studies demonstrated that systemic expositions to estrogens are associated with a significantly increased risk of recurrence of breast cancer (HR 3.5) or new primary development, thus contraindicating the use of this strategy in breast cancer survivors [49].

Although the role of systemic HT is well known, the debate about the safety of its administration is still open. When asked, 71% of oncologists mentioned that the main reason not to prescribe vaginal estrogens is the concern about the potential systemic absorption and the consequent possibility of increased risk of recurrence [15].

Dew et al., in a small cohort study, and Le Ray et al., in a case-control study, of 340 patients with early BC, investigated the BC recurrence risk associated with the use of vaginal estrogen therapy [50,51]. In both works, there is no evidence of an increased risk of recurrence.

In a Danish observational study, a cohort of postmenopausal women with early-stage invasive estrogen receptor-positive nonmetastatic BC who received no treatment or 5 years of adjuvant endocrine therapy were followed over time to evaluate the recurrence and mortality that each had and according to VET, MHT, or no therapy received. The authors concluded that in postmenopausal BCS, neither VET nor MHT was associated with an increased risk of recurrence or mortality. A subgroup analysis revealed an increased risk of recurrence, but not mortality, in patients receiving VET with adjuvant aromatase inhibitors [52].

However, the studies had several limitations, including a small sample size and a short follow-up.

Therefore, it remains unclear whether VET or MHT is safe for women treated for BC [53,54].

Among women without a history of BC, a meta-analysis from the Collaborative Group on Hormonal Factors in Breast Cancer reported an increased risk of primary BC among women treated with MHT compared with never-users, whereas VET was not associated with an increased risk of BC [55,56].

When discussing circulating estrogen levels, it is important to highlight that there is no validated safe threshold, but it is commonly assumed that post-menopausal levels must be achieved. There is not a shared opinion about what specific estradiol or estrone levels should raise concern for breast cancer survivors. In postmenopausal women not receiving hormone therapy, average estradiol levels reach 14.1 pg/mL and estrone levels 27.5 pg/mL; it is unclear if keeping values within the typical postmenopausal range is sufficient to decrease the risk of BC recurrence [57].

Estradiol systemic absorption is dose-dependent, and it is influenced by dose, formulation, and positioning in the vagina. During the last few years, several clinical trials were designed to analyze the pharmacokinetics of different vaginal devices to assess safety and efficacy [58].

Eugster-Hausmann et al. conducted a study in which 58 postmenopausal women received 10 mg or 25 mg estradiol vaginal tablets. The trial proved that systemic estradiol absorption with the 10 mg tablet was lower when compared with the 25 mg tablet. Furthermore, after 1 year of treatment with the 10 mg vaginal tablet, levels of estrogen in the body were within the menopausal range (2.44 to 12.08 pg/mL), indicating minimal absorption and a potentially safe alternative in breast cancer survivors receiving AIs [59].

Other treatments that have been considered include intravaginal androgens, based on the concept that administration may act on androgen receptors that have been identified in the wall of the vagina. Local administration of testosterone at the vaginal level appeared to trigger the activation of estrogen and androgen receptors in the vaginal epithelial layers without activating estrogen receptors in other tissues due to the lack of aromatase at this level. Testosterone can induce proliferation of the vaginal epithelium, but the conversion of testosterone to estrogen is blocked by Ais, and thus, it may be effective in reversing the atrophic changes without increasing circulating estrogen levels and compromising aromatase inhibitor therapy [60].

Some recent studies are evaluating the effects of a new androgen, dehydroepiandrosterone (DHEA), in the treatment of vaginal dryness in patients with previous BC [61]. Labrie et al. (2017) in their randomized clinical trial demonstrated the efficacy of prasterone (administered intravaginally at 0.50% 6.5 mg) in postmenopausal women suffering from moderate to severe dyspareunia due to vulvovaginal atrophy. In particular, an average 35.1% decrease over placebo in the percentage of parabasal cells (*p* < 0.0001), an average 7.7% increase in the percentage of superficial cells (*p* < 0.0001), and a mean 0.72 pH unit decrease in vaginal pH (*p* < 0.0001) were observed. Moreover, a very positive evaluation was obtained on the acceptability of the technique of administration of the insert, whereas the male partners reported a very positive evaluation of the changes observed in their sexual partners [62].

At present, this androgen appears to be the only one approved by the FDA for the treatment of GSM in the form of prasterone, the synthetic analog of DHEA.

Barton et al. conducted a phase III randomized clinical trial that evaluated vaginal administration of DHEA at the doses of 3.25 or 6.5 mg compared with plain moisturizer in postmenopausal women with a history of breast (97%) or gynecologic cancer who could be receiving HT (56%). Peripheral blood sample analysis showed that circulating estradiol was significantly increased in those receiving 6.5 mg/d DHEA but not in those receiving 3.25 mg/d DHEA or AI therapy. In both arms, mean estradiol concentrations remained lower than 5 pg/mL. Assuming that hormone concentrations, even though slightly increased, remain in the lowest half or quartile of the postmenopausal range, the FDA has approved vaginal DHEA for the treatment of GSM [63]. However, the prasterone technical data sheet includes a warning against its use in BCS.

There are no studies directly comparing vaginal DHEA to vaginal estrogens in terms of efficacy or studies comparing systemic hormone levels; therefore, there can be no recommendation for one over the other in BCS. Vaginal testosterone cream and an estradiol-releasing vaginal ring also proved to safely improve GSM symptoms in BCS. A study by Witherby et al. supported the safety of vaginal testosterone at the dose of 150 mg or 300 mg daily for treating vulvovaginal atrophy in patients with breast cancer receiving AI therapy, not detecting any significant elevation in serum estradiol levels (<8 pg/mL) at either dose of testosterone [64].

Melisko et al. conducted a randomized phase II trial evaluating the safety and efficacy of intravaginal testosterone cream or a vaginal ring for 12 weeks in postmenopausal women receiving AIs who had symptoms of vulvovaginal atrophy. The intervention was considered unsafe if more than 25% of patients had elevations in serum estradiol greater than 10 pg/mL and at least 10 pg/mL above baseline after treatment initiation. Both interventions met the primary safety end point and improved vaginal atrophy, sexual interest, and dysfunction [65].

In conclusion, according to the American College of Obstetricians and Gynecologists (ACOG) recommendations, the first-line approach to managing GSM in BCS receiving HT is non-hormonal options, but when refractory or severe symptoms occur, the use of vaginal estradiol or DHEA can be considered a safe option to be offered to patients to improve their quality of life [54,57].

Novel emerging approaches, including SERMs, TSECs, estriol, and neurokinin B-inhibitors, showed promising activity in managing GSM symptoms with a hypothetical neutral activity on BC. Further investigations in women affected by BC are required to assess the safety and efficacy of these compounds [66].

Another emerging therapy for the treatment of VVA is the vaginal laser. The first studies were performed using a CO_2_ fractional laser approved by the Food and Drug Administration (FDA) as a therapy for GSM [67]. The mechanism of action of the laser consists in stimulating progressive neo-collagenases and fibroblast activation with the production of new trabecular-type collagen. The active fibroblasts in the lamina propria also determine an increase in elastin. The use of reverse transcriptase with PCR allowed to demonstrate of a significant increase of pro-collagen mRNA and interleukin-b and TGF-b1, with epithelial reactivation and cellular differentiation [68].

Subsequently, the Erbium laser was introduced, which is better at promoting vascularization of the vaginal mucosa due to a direct correlation between energy density and penetration depth. In fact, Erbium has a non-ablative photothermal effect with less discomfort and less blood loss capable of improving the local accumulation of glycogen and promoting the restoration of an adequate local pH [69]. Another type of laser that finds application in the treatment of vulvovaginal atrophy is the CO_2_ laser. The CO_2_ laser, unlike the Erbium laser, is able to act on the more superficial collagen, improving the vascularization of the tissues in order to obtain better integrity and elasticity of the tissues. Additionally, CO_2_ lasers have been shown to improve stress urinary incontinence and vaginal prolapse, as well as vaginal dryness and dyspareunia. A recent meta-analysis by Salvatore et al. conducted in 2022 analyzed the CO_2_ laser therapy efficacy for the treatment of GSM, no matter whether the patients were postmenopausal or BCS. They demonstrated that in all scores (FSFI, WHIS, and VMV scores), laser CO_2_ therapy has led to a significant reduction in VVA and/or GSM symptoms. Its application showed a beneficial safety profile, and no major adverse events were reported [70].

However, the available data are short-term, and the efficacy, as well as the safety of repeated applications, are unclear. Furthermore, CO_2_ laser treatment is very expensive and a procedure that is not yet widely performed by gynecologists; therefore, access to this method may be limited [71].

In 2018, Becorpi et al., in a prospective study, analyzed a sample of 20 patients for one month, treating them with CO_2_ laser. They demonstrated that the laser caused changes both at a biomolecular and morphological level in the tissues by activating anti-inflammatory mechanisms [72]. Subsequently, two more prospective studies were conducted, the first by Quick et al. in 2022 and the second and most recent by Mension et al. in 2023. Both studies demonstrated that CO_2_ laser is safe and effective, respectively, after 6 months and 2 years of follow-up in patients with previous BC [73,74]. Squilini’s group retrospectively analyzed a cohort of 45 BCS patients, comparing them to 90 control group patients for one year. They found that fractional CO_2_ vaginal laser led to a long-term improvement in GSM symptoms, even in BCS [75].

The only RCT in the literature that analyzes laser therapy in patients with GSM is the one conducted in 2021 by Gold and Collab. These were analyzed for 3 months on a group of 43 patients treated with Erbium laser or local hyaluronic acid. Both treatments were effective for patients with previous breast cancer [76]. Even if the effectiveness of vaginal lasers using both CO_2_ and Erbium is confirmed in most studies, there are no studies that demonstrate their real long-term effectiveness. We have summarized the various laser therapies in Table 2.

The last family of drugs that we are going to examine is selective estrogen receptor modulators (SERMs). It is a class of drugs that have activity on estrogen receptors, the most representative of which in the therapy of GSM in BCS are Tibolone, Bazedoxifene, and Ospemifene.

The LIBERATE (The Livial Intervention following Breast Cancer; Efficacy, Recurrence, and Tolerability Endpoints) study was designed to establish the safety of tibolone in women operated on for breast cancer suffering from severe menopause symptoms. The study, a double-blind, non-inferiority, multicenter RCT conducted on 3148 postmenopausal women (mean age 53 years) with a history of surgically treated breast cancers, compares therapy with tibolone (2.5 mg/day) versus placebo in terms of breast cancer recurrences. After a median follow-up of 3.1 years, a statistically significant increase in the incidence of tumor recurrence was observed in the tibolone group (237 cases vs. 165 in the placebo group; HR 1.4; CI 95% 1.1–1.7) [77].

In the literature, there are two RCTs that evaluated the activity of bazedoxifene combined with estrogens of animal origin. In the first study, healthy, postmenopausal, non-hysterectomized women (n = 652) with symptoms of moderate to severe vulvar/vaginal atrophy treated with a drug or placebo were considered. It was seen that the drug treatment had a decidedly positive efficacy in the treatment of vulvar atrophy and consequently also on the sexual hygiene of the patients, improving their quality of life. However, bazedoxifene alone, not combined with estrogen, has not been shown to improve symptoms [78].

The second study presented by Kagan et al. showed similar results in another RCT; however, neither study evaluated the safety of the drug in patients with previous breast cancer, so at present, there are no recommendations for the administration of this drug [79].

Ospemifene is a drug belonging to the class of SERMs that has the ability to act on various organs: an agonist action on the brain, vaginal epithelium, and bone; an anti-estrogenic activity on the breast; and it would appear to have no activity on the endometrium or the cardio-circulatory system.

The activity on the vaginal epithelium favors epithelial thickening, favoring its lubrication.

There are no clinical data demonstrating that ospemifene would increase the risk of BC; indeed, its anti-estrogenic activity in the breast could be protective against a possible recurrence. However, the follow-up periods of these studies were too short to conclude the long-term effects of ospemifene [80]. Barton et al. (2017) conducted a three-arm randomized controlled trial on patients with previous breast cancer or gynecological patients using DHEA gel at two different doses or a placebo. What they found is that daily use of the gel has the ability to improve vaginal dryness after 12 weeks of treatment but is unable to impact sexual function [63]. Subsequently, in a randomized double-blind study, Davis’ group demonstrated that the administration of testosterone locally by ointment or cream is able to promote sexual function compared to a placebo in a group of BCS patients [66].

The latest randomized study in the literature of 2020 is the result of the activity of Hirschberg’s group. This is a large multicenter prospective randomized double-blind placebo-controlled phase II study, which analyzed a group of 61 patients treated with local estrogens for 12 weeks. What was achieved was that there were no significant differences in FSH between groups (*p* = 0.104) with a slight increase in LH in the treatment group. No changes in E1 and E2 were observed in estrogen-treated patients, other than a slight transient increase in E3 within the first 3 weeks. It is therefore possible to state that administration of 0.005% estriol gel is safe in BCS receiving NSAI and provides clinical improvement of vaginal symptoms and signs of VVA [81]. The observational study proposed by Cold et al. included a nationwide cohort of postmenopausal women diagnosed between 1997 and 2004 with early-stage invasive estrogen receptor-positive nonmetastatic BC who received no treatment or 5 years of adjuvant endocrine therapy. We evaluated the mortality and risk of relapse associated with VET and MHT use versus no use using multivariable models adjusted for potential confounders. The study concluded that in postmenopausal women treated for early-stage estrogen receptor-positive BC, neither VET nor MHT was associated with an increased risk of recurrence or mortality. A subgroup analysis revealed an increased risk of recurrence but not mortality in patients trained on adjuvant aromatase inhibition [52].

Below, in Table 3, all the local hormone treatments are listed.

In conclusion, we deem it appropriate to say a few words about patients with non-luminal BC. Patients with non-luminal BC (HER2-positive and triple-negative) are not candidates for adjuvant HT because there has long been a consensus that they are completely independent of the estrogen signaling pathway. Chemotherapy and anti-HER2 agents are the gold standards of treatment.

Even if they do not receive AI or tamoxifen, women with non-luminal BC may experience GSM as a consequence of chemo-induced ovarian failure or age-related menopause. At first glance, the use of estrogen compounds in this population could be considered absolutely safe; however, there is no conclusive evidence in the literature to support this hypothesis [82].

According to Kenemans et al. [83], there is no significant adverse effect of HRT in the subgroup analysis of patients with ER-negative BC exposed to HRT, whereas Holmberg et al. [48] showed an increased, but not statistically significant, risk of recurrence.

Preclinical evidence has also recently demonstrated that estrogen and progesterone can circumvent the absence of their respective traditional nuclear receptors in TNBC and play an active role in cancer progression [84]. Most of the evidence comes from retrospective cohorts or subgroup analyses of studies designed primarily for luminal BC. In conclusion, there is insufficient evidence to allow the use of systemic HRT for GSM in patients with TNBC. In our daily clinical practice, the treatment of symptoms in this subset of patients should be guided by the same guidelines approved for luminal BC, preferring non-hormonal or local therapy to systemic administration [85].

## 4. Conclusions

In BCS patients, vaginal atrophy is one of the main causes of reduced quality of life, both for individuals and couples. Patients with mild atrophy can be treated with therapy based on non-hormonal substances; however, these medicines need a long time to act and are relatively effective. In patients’ refractory to non-hormonal therapy, local, low-dose estrogen administration has been shown to be the most effective treatment. Although there is strong skepticism about the use of hormone therapy in patients with previous breast cancer, this seems to be the most effective therapy to date in restoring adequate vaginal tropism. Some studies suggest a possible increase in serum estrogen levels, which may lead to an increased risk of BC recurrence. In the new guidelines, further new therapeutic strategies with mechanical and non-pharmacological action are considered, such as the vaginal laser. However, more studies are needed to evaluate. More studies are needed to evaluate the effectiveness of these new therapies.

## Figures and Tables

**Table 1:** Non-hormonal treatments.

Study	Year	Study Design	Follow Up	Median Age	n.	Participants	Intervention	Primary Outcome	Hormonal BC Therapy	Receptor Status (ER +)	Results	Conclusions
Carter et al. Supportive Care in Cancer	2021	Single-arm, prospective longitudinal trial	6 months	55 years old	101	HR+ breast cancer treated with AI or HR+ endometrial cancer treated with surgery and postoperative radiation.	HLA daily for the first 2 weeks, and then 3×/week until weeks 12–14; dosage was then increased to 5×/week for non-responders.	VAS, VuAS, FSFI, MSCL	AI	All	VAS/VuAS scores significantly improved at all assessment points (all *p* < 0.001). MSCL scores similarly improved (all *p* < 0.001). FSFI scores significantly improved from T1 to T2 (*p* < 0.03), T3 (*p* < 0.001), and T4 (*p* < 0.001). Severe vaginal pH (>6.5) decreased from 26% at T1 to 19% at T4 (*p* = 0.18).	HLA moisturization improved the vulvovaginal health/sexual function of cancer survivors. While HLA administration 1–2×/week is recommended for women in natural menopause, a 3–5×/week schedule appears to be more effective for symptom relief in cancer survivors.
Keshavarzi et al. Supportive Care in Cancer	2019	Randomized triple-blind study	8 weeks	43.2 years old	96	Patients with diagnosed BC on adjuvant therapy with TAM.	Vit D (1000 UI), Vit E (1 mg), or placebo suppositories	To investigate the effect of vitamin D and E vaginal suppositories on vaginal atrophy in women with breast cancer receiving tamoxifen.	TAM	-	Increase in VMI of the groups receiving Vit E and D compared with placebo (*p* < 0.001); vaginal pH and subjective symptoms reduced in the two groups compared with placebo.	These data support that vitamin D and E vaginal suppositories were beneficial in improving vaginal atrophy in women with breast cancer receiving tamoxifen.
Chatsiproios et al. PlosOne	2019	Open, prospective, multicentric, observational study.	28 days	52 years old	128	Patients with diagnosed BC managed with chemotherapy or hormonal therapy.	Oil-in-water emulsion during 28 days.	Subjective Symptoms; safety and tolerability.	-	-	The difference in symptom frequency before-after the treatment was significant (*p* < 0.0001).	This treatment seems to improve VVA symptoms with a short treatment.
Hersant et al. Menopause	2018	Prospective, comparative(before/after) pilot study	6 months	60.8 years old	20	Patients with diagnosed BC	A-PRP and evaluated at 0,1,3 and 6 months.	Evaluated vaginal mucosa changes using Vaginal Health Index.	-		Significant increase 10.7 to 20.75 (*p* < 0.0001) at 6 months.	A-PRP improves vaginal mucosa within 6 months of treatment according to VHI criteria.
Marschalek et al. Breast Care	2017	Randomized Controlled Trial, Double Blinded Pilot study	2 weeks	59 years old	11 Lactobacillus 11 placebo	Patients with diagnosed BC managed with chemotherapy or hormonal therapy	Vaginal lactobacillus capsules vs. placebo	Nugent score	-		Not reported. Differences between groups: 4.73 vs. 4.0 (*p* = 0.038).	Lactobacillus improves microbiota in BCS.
Juliato PT et al. Climacteric	2016	Randomized Trial	30 days	50.5 years old	25 Polyacrylic acid 27 lubricant	Patients with diagnosed BC treated with tamoxifen.	Polyacrylic acid vs. Lubricant	FSFI	-	-	Both showed improvement. Acid: 96 to 24% (*p* = 0.0001). Lubricant: 88.9 to 55.6% (*p* = 0.0027).	Polyacrylic acid was superior to lubricant.
Goetsch et al. Journal of Clinical Oncology	2015	Randomized Controlled Trial, Double Blinded	4 weeks	56.6 years old	23 Lidocaine 23 Saline	Patients with diagnosed BC	4% aqueous lidocaine vs. placebo 3 min before vaginal penetration.	Pain (VAS scale)	-	-	Significative differences between groups (*p* < 0.007).	It is a safe option for painful intercourse.
Juraskova et al. J Sex Med	2013	Phase I/II Prospective Study	26 weeks	51 years old	16	Patients with diagnosed BC treated with AI	Pelvic floor muscle (PFM) relaxation exercises twice/day, polycarbophil-based vaginal moisturizer three times/week, and olive oil as a lubricant during intercourse.	Dyspareunia, sexual functioning, quality of life, distress, and pelvic floor muscles (PFMs) functioning	All	-	OVERcome resulted in significant improvements in dyspareunia, sexual function, and quality of life over time (all *p* < 0.001). PFM relaxation training was reported to be effective (*p* < 0.001). Maximum benefits were observed in week 12.	Efficacy in improving dyspareunia and sexual function following breast cancer was demonstrated.
Lee YK et al. ACOG	2011	Randomized Controlled Trial, Double Blinded	12 weeks	45.8 years old	44	Patients with diagnosed BC managed with chemotherapy or hormonal therapy	pH balanced gel vs. placebo for 12 weeks	Vaginal dryness and dyspareunia	-	-	There was a significant difference in the variable dryness with pain (*p* = 0.001), and in the variable dyspareunia (*p* = 0.04).	A vaginal pH-balanced gel could relieve vaginal symptoms.

**Table 2:** Laser therapy.

Study	Year	Study Design	Follow Up	Median Age	n.	Participants	Intervention	Primary Outcome	Hormonal BC Therapy	Receptor Status (ER +)	Results	Conclusions
Mension et al. JAMA Network	2023	Prospective double-blind sham-controlled Randomized Clinical Trial	6 months	52.6 years old	72	BC patients on adjuvant AI therapy	5 monthly sessions of fractional CO_2_ laser therapy (CLT) or sham laser therapy (SLT).	FSFI, VHI, Objective improvement	-	-	Both groups showed improvement in FSFI, but there was no significant difference in subjective and objective outcomes between CLT and SLT groups. Tolerance to treatment was significantly lower in the CLT group than in the SLT group.	Vaginal laser treatment was found to be safe and effective after 6 months of follow-up.
Quick et al. J of Clin Med	2022	Prospective study	2 years	59.3 years old	33	Patients with diagnosed BC on adjuvant therapy (AI or trastuzumab)	3 sessions of micro ablative CO_2_ laser 30–45 days apart	Long-term efficacy: VAS, FSFI, UDI	AI or trastuzumab	-	No statistically significant difference in VAS score, FSFI, and UDI score between 4 weeks follow-up and 2 years follow-up.	Breast cancer survivors treated with fractional CO_2_ laser therapy have sustained improvement in sexual function two years after treatment completion, suggesting potential long-term benefits.
Gold et al. Maturitas	2021	Randomized controlled trial	3 months	54 years old	43	Patients with diagnosed BC	2 sessions of Erbium YAG Laser therapy one month apart or vaginal hyaluronic acid, daily for 10 days then 3 times/week for three months	VHIClinical and sexual improvement	-	-	VHI improved significantly in both groups (*p* = 0.001) with no differences between treatment groups (*p* = 0.232). Clinical and sexual improvements in both groups without differences.	Both vaginal hyaluronic acid and Ervium Yag Laser are effective to treat GSM in BCS.
Squillini et al. The Breast J	2021	Retrospective study	12 months	59.5 years old	45 BC patients 90 controls	Patients with diagnosed BC	3 sessions of fractional micro ablative CO_2_ laser every 30 days	VHI, VVHI, dyspareunia, and vaginal dryness	Previous endocrine therapy 51.4% Current endocrine therapy 48.6%		BCS are most likely to present severe GSM symptoms compared with the comparison group. VHI and VVHI were improved in both groups.	Fractional CO_2_ vaginal laser leads to a long-term improvement in GSM symptoms, even in BCS.
Angioli et al. Intern J of Gynecol Cancer	2020	Retrospective multicentric study	3 months	53 years old	165	Patients with diagnosed Breast, Ovary, Uterus, or Cervical Cancer	3 sessions of fractional micro ablative CO_2_ laser 30 days apart	Objective and subjective improvement	-		Dryness improved by 66%, dyspareunia improved by 59%, burning improved by 66%, pain at the introitus improved by 54%, and itching improved by 54%.	An effective strategy in the management of the symptoms of genitourinary syndrome in post-menopausal women and survivors of gynecological cancer.
Areas et al. Menopause	2019	Open, prospective study	4 months	53.7 years old	24	Patients with diagnosed BC	3 sessions of Vaginal Erbium YAG Laser every 30 days.	Clinical and Sexual improvement: VAS scale—Objective VHI.	-	-	Improvement in clinical and sexual scores. Last follow-up vs. Basal VHI: significant reduction (*p* < 0.001).	The treatment seems to improve sexual function and vaginal atrophy.
Pearson et al. Breast Cancer Research and Treatment	2019	Single-arm pilot study Before-After Study	12 weeks	56 years old	26	Patients with diagnosed BC	3 sessions of Fractional Microablative CO_2_ Laser every 30 days.	Clinical and Sexual improvement: VAS scale and FSFI	96% Hormonal Therapy	-	Improvement in VAS after 3 sessions (*p* < 0.001). Improvement in FSFI after 3 sessions (*p* < 0.01).	The treatment seems to improve sexual function and vaginal atrophy.
Quick et al. Supportive Care in Cancer	2019	Single-arm feasibility study	1 month	57.4 years old	64	Patients with diagnosed BC on adjuvant therapy	3 sessions of micro ablative CO_2_ laser 30–45 days apart	Feasibility, adverse events	AI 68%	63% ER/PR+/Her2-	No women presented serious adverse events. VAS, FSFI, and UDI improved to follow-up.	Fractional CO_2_ laser treatment for breast cancer survivors is feasible and appears to reduce GSM symptoms across treatment and follow-up.
Becorpi et al. Lasers in Medical Science	2018	Prospective study	1 month	58.2 years old	20	Patients with diagnosed BC	2 sessions of fractional CO_2_ laser	Objective and subjective improvement, Microbiome analysis, Vaginal cytokine analysis	-	-	Statistically significant improvement for VHI, FSFI. Higher levels of IL-18, CTACK LIF, M-CSF, and IL-17. The Shannon diversity index H and equitability comparison before and after treatment did not yield any statistically significant results.	Efficacy of laser on GSM in BCS due to the biochemical and morphological changes of the epithelial vaginal cells which are associated with the expression of specific cytokines involved, in the anti-inflammatory process. Maintenance of a positive local balance is able to favor the colonization of commensal microorganisms.
Pagano et al. Menopause	2018	Observational retrospective study	3 months	44 years old	82	Patients with diagnosed BC	3 sessions of Fractional Microablative CO_2_ Laser every 30 days.	Clinical improvement: VAS scale	74% Hormonal adjuvant treatment; 61% AI; 39% TMX	-	Improvement in VAS after 3 sessions (*p* < 0.001).	The treatment seems to be effective.
Mothes et al. Journal of Cancer Research and Clinical Oncology	2018	Retrospective study	6 weeks	71 years old	16	Patients with diagnosed BC and surgery for pelvic organ prolapse	1 session of Vaginal Erbium YAG Laser.	Clinical improvement: VAS scale	-	-	Last follow-up vs. Basal VHI: significant reduction (*p* = 0.01).	The treatment seems to be effective.
Gambacciani et al. Menopause	2017	Pilot study Before-After study		50.8 years old	43	Patients with diagnosed BC	3 sessions of Vaginal Erbium Laser every 30 days.	Clinical improvement: VAS scale—Objective VHI.	-	-	Last follow-up vs. Basal VAS: significative reduction (*p* < 0.01). Last follow-up vs. Basal VHI: significative reduction (*p* < 0.01).	The treatment seems to be effective.
Pagano et al. Menopause	2016	Observational retrospective study	3 months	42 years old	26	BC patients on adjuvant TMX or AI therapy	3 sessions of Fractional Microablative CO_2_ Laser every 30 days.	Clinical improvement: VAS scale—Objective VHI.	All	All	Significant improvement of clinical variables.	The treatment seems to be effective and with good tolerance.
Pieralli et al. Arch Gynecol Obstet	2016	Prospective Before-After study	11 months	53.3 years old	50	Patients with diagnosed BC	3 sessions of Fractional Microablative CO_2_ Laser every 30 days.	Clinical improvement: VAS scale—Objective VHI.	4% AI; 40% TMX; 56% Not adjuvant therapy	-	Improvement in VAS after 3 sessions (*p* < 0.0001).	The treatment seems to be feasible and effective.

**Table 3:** Local hormonal treatments.

Study	Year	Study Design	Follow Up	Median Age	n.	Participants	Intervention	Primary Outcome	Hormonal BC Therapy	Receptor Status (ER +)	Results	Conclusions
Cold et al. J Natl Cancer Inst	2022	Observational Cohort Study	20 years	61 years old	1957 VET 133 MHT (+/− VET) 6371 never-user	BC patients on adjuvant AI or TAM or no therapy	“VET”: “MHT”: “never-user”	Recurrence Mortality	TAM, AI, both, or none	All	VET risk of recurrence was similar to never-users (HR 1⁄4 1.08, 95% CI 1⁄4 0.89 to 1.32). The use of VET in patients who received AI was associated with an elevated risk of recurrence (HR 1⁄4 1.39, 95% CI 1⁄4 1.04 to 1.85). The cumulative incidence of recurrence was 19.2% in never-users, 15.4% in VET users, and 17.1% in users of MHT. Never-users of VET or MHT had an absolute 10-year overall survival of 73.8% compared with 79.5% and 80.5% among the women who used VET or MHT, respectively.	VET or MHT is not associated with an increased risk of recurrence or mortality. In patients treated with VET and adjuvant AIs (but not TAM or no endocrine adjuvant therapy), increased risk of recurrence but not mortality. Overlapping overall survival in the three groups.
Streff et al. Supportive Care in Cancer	2021	Prospective trial	16 weeks	55 years old	8 treated 6 controls	BC patients on adjuvant AI therapy	“Estring”: estradiol 2 mg ring placed vaginally over 90 days	Estradiol serum level; clinical improvement	AI (anastrozole, letrozole or exemestane)	All	Estradiol level was <10 pg/mL in all patients; the week-4-estradiol level was >10 pg/mL in 6 cases (75%) but decreased to <10 pg/mL by week 14.	No significant change in serum estradiol level in BCS treated with AI from baseline to week 16.
Hirschberg et al. J North American Menopause Society	2020	Phase II prospective randomized double-blind placebo-controlled multicentric trial	12 weeks	59 years old	61	BC patients on adjuvant AI therapy	Estriol gel 50 mg daily for 3 weeks and twice weekly for 6 weeks vs. placebo	FSH, LH, estrogens serum levels; clinical improvement	AI (anastrozole or letrozole) + evtl TAM + evtl LHRH agonist	All	No significant differences in FSH between groups (*p* = 0.104); a slight rise of LH in the treatment group; no change in E1 and E2; slight E3 rise transiently within the first 3 weeks; clinical improvement. A significant difference in favor of the TST group.	0.005% estriol gel preparation is safe in BCS receiving NSAI and provides a clinical improvement in vaginal symptoms and signs of VVA.
Davis et al. J Clin Endocrinol Metab	2018	Double-blind randomized placebo-controlled trial	26 weeks	56.4 years old	44	BC patients on adjuvant AI therapy	TST cream for 26 weeks 300 ng vs. placebo	FSFI	AI		A significant difference in favor of the TST group.	TST improves sexual function compared to placebo.
Barton et al. Support Care Cancer	2017	Three-arm randomized controlled trial	12 weeks	57.4 years old	353	BC or gynecological patients on adjuvant TAM or AI therapy	DHEA gel 3.25 mg vs. DHEA gel 6.5 mg vs. Placebo, administred daily	FSFI	AI or TAM		Overall clinical improvement in all arms but not significantly different between arms. Women in the DHEA arms reported significant improvement in the sexual health measure. DHEA 6.5 mg, improved symptoms more quickly, with a significant difference at 8 weeks.	Daily use of a vaginal moisturizer has the ability to improve vaginal symptoms over 12 weeks but may not sufficiently positively impact overall sexual function.
Melisko et al. JAMA Oncology	2016	Randomized non-comparative study	12 weeks	56 years old	76	BC patients on adjuvant AI therapy	“Estring” Estradiol ring 7.5 ng vs. TST cream at 1% 1.5 mg/week	E2 serum levels; VHI	AI	All	Transient E2 rise in the estradiol group and 12% in the TST group. Persistent E2 rise of 0% in the estradiol group and 12% in the TST group. Sexual improvement in both groups.	Transient increase in E2 with “Estring”. Meets the primary safety endpoint.
Dahir et al. Sexual Medicine	2014	Pilot Study	8 weeks	59.7 years old	13	BC patients on adjuvant AI therapy	TST cream for 28 days 300 ng	FSFI	AI (anastrozole, letrozole or exemestane)	92% ER+ 84% PR+	Clinical improvement.	Improvement in FSFI scores.
Donders et al. Breast Cancer Res Treat	2014	Open-label bicentric phase I pharmacokinetic study	12 weeks	57 years old	16	BC patients on adjuvant AI therapy	Estriol 0.03 mg + Lactobacillus	E2 (estradiol), E1(estrone), E3(estriol) serum levels; clinical improvement	AI	All	No change in E1 and E2. Small transient increase in E3. Clinical improvement in 100%.	E3 + Lactobacillus is safe in BC patients and improves symptoms.
Wills et al. Journal of Oncology practice	2012	Prospective clinical study	12 weeks	60 years old (BCS) 68 years old (Controls)	24 therapy 24 controls	BC patients on adjuvant AI therapy or SERM	(14 p) Vaginal Estradiol tablet 25 mcg daily for 14 days, then twice a week (10 p) “Estring” Estradiol ring	E2 serum levels	AI or SERM	All	E2 was significantly greater in the VET group. Mean E2 levels of controls: 3.72 pmol/L Mean E2 levels 12 h post tablet insertion: 76 pmol/L (significantly higher than controls *p* < 0.001). Mean E2 levels 60 days post ring insertion: 15 pmol/L (*p* < 0.014).	VET increases E2 levels. Should be used with caution.
Le Ray et al. Breast Cancer Res Treat	2012	Retrospective cohort study	3.5 years	63.7 years old	13.479	917 BC recurrence patients 8885 Controls	Vaginal estrogen cream and tablets	Recurrence	TAM or AI	All	Recurrence RR 0.78 (95% CI: 0.48–1.25).	VET is not associated with an increase in BC recurrence in patients treated with TAM or AI.
Whiterby et al. The Oncologist	2011	Phase I/II pilot study	8 weeks	56 years old	21	BC patients on adjuvant AI therapy	TST cream for 28 days 300/150 ng	E2 and TST levels; clinical improvement	AI	-	E2 levels remained suppressed. Clinical improvement.	Clinical efficacy and tolerance.
Pfeiler et al. Climateric	2011	Prospective before-after analysis	4 weeks	65 years old	6	BC patients on adjuvant AI therapy	“Ovestim” 0.5 mg vaginal estriol daily for 2 weeks	Clinical improvement, serum estradiol level	Anastrozole	All	Clinical improvement in 83%, with no increased serum estradiol levels at 2 weeks.	VET improves clinical VVA and appears to be safe.
Biglia et al. Gynecological Endocrinology	2010	Prospective study	12 weeks	54 years old (VET) 46 years old (Replens)	31	BCS in menopause: 18 receiving VET 8 receiving moisturizers	Estriol 0.25 mg Estradiol 12.5 ng 2.5 g Replens	Clinical Improvement, Objective vaginal mucosa evaluation Estradiol, FSH, and LH serum levels	TAM and GnRH	72% Estriol 87% Estradiol	Improved symptoms in both VET groups (*p* = 0.02, *p* = 0.01) No change in estradiol, increased FSH and LH.	VET is effective in improving symptoms and objective evaluation. An increase in FSH and LH may indicate a systemic estradiol effect.
Kendall et al. Annals of Oncology	2005	Prospective before-after analysis	12 weeks	52 years old	7	BC patients on adjuvant AI therapy	“Vagifem”: Estradiol 25 mg daily for 2 weeks	Estradiol serum levels	AI (anastrozole, letrozole or exemestane)	-	2 weeks: 83% estradiol rise 10 weeks: 66% estradiol rise.	Serum estradiol levels increased short term.
Dew et al. Climateric	2003	Retrospective Cohort Study	5.5 years	53.8 years old	69	69 BC patients with VVA 1403 BC patients without VVA	Estriol 0.5 mg cream and pessaries (n = 33) Estradiol 25 mcg tablets (n = 33)	Disease-free Interval Recurrence	48% TAM	Positive in 12/33 (36%)	DFI HR = 0.57 (95% CI: 0.29–1.58, *p* = 0.28). 6 (9%) vs. 330 (22.4%).	VET does not seem to be associated with increased RR of BC.

## Data Availability

Data sharing is not applicable.
